# Clinical significance of lymphocytopenia in patients hospitalized with pneumonia caused by influenza virus

**DOI:** 10.1186/s13054-019-2608-1

**Published:** 2019-10-29

**Authors:** Valeria Bellelli, Gabriella d’Ettorre, Luigi Celani, Cristian Borrazzo, Giancarlo Ceccarelli, Mario Venditti

**Affiliations:** Department of Public Health and Infectious Diseases, University of Rome Sapienza, Viale del Policlinico 155, Rome, Italy

Recently, lymphocytopenia has been evaluated as an independent biomarker of mortality in hospitalized patients diagnosed with community-acquired pneumonia (CAP) [1, 2]. In these studies, patients recruited were affected by CAP due to different etiology, and no specific differences have been observed in viral and bacterial etiology distribution. On the other hand, in influenza virus infection, lymphocytopenia has been identified as a risk factor for bacterial superinfections [3], determining a worse prognosis. We would like to evaluate the potential role of lymphocytopenia as a prognostic factor in patients with pneumonia caused by influenza virus.

For this purpose, we performed a retrospective, observational study on patients hospitalized in a hospital in Rome with pneumonia due to influenza virus. Between January and April 2019, we observed 38 patients with either CAP (29 patients) or hospital-acquired pneumonia (9 patients) due to influenza virus (defined by the presence of fever, symptoms and signs of pneumonia syndrome, new onset of pulmonary infiltrates on chest X-rays or CT scans, and influenza virus detection on respiratory specimens). Consistent with already published data [4], the rate of nosocomial infections found was high and requires substantial improvement of early diagnosis and infection control strategies. Focusing on the lymphocyte count at the onset of infection, with the adoption of a previously reported cutoff value of 724 lymphocytes/μl [2], 23 patients were considered as affected by lymphocytopenic influenza virus pneumonia (L-IP) and 15 by a non-lymphocytopenic influenza virus pneumonia (NL-IP). As shown in Table 1, in comparison with NL-IP, patients with L-IP were more commonly affected by COPD (*p* = 0.046), they more frequently required admission to the intensive care unit (*p* = 0.002) and invasive mechanical ventilation (*p* = 0.031) and presented a higher SOFA score at the time of diagnosis (*p* = 0.004); they also experienced more frequently secondary bacterial and fungal pulmonary superinfections. As shown in Table 1, secondary bacterial pathogens were often multiple resistant nosocomial organisms as carbapenem-resistant *Acinetobacter baumannnii* and *Corynebacterium striatum* and methicillin-resistant *Staphylococcus aureus*. Notably, fungal pathogens were represented not only by *Aspergillus fumigatus* but also by *Pneumocystis jiroveci*. If the former association is well known, to our knowledge, only one case report has been published on *P. jiroveci* superinfection in an immunocompetent host affected by influenza virus [5]. Analyzing only the influenza CAP patients, the results were similar: SOFA score at the time of diagnosis was higher in patients with L-IP (*p* = 0.013) and they experienced more frequently respiratory failure requiring oxygen support (*p* < 0.001) and IMV (*p* = 0.045) (Table 1). Moreover, all the episodes of superinfection were experienced by lymphocytopenic patients.

**Table 1:** Baseline characteristics, severity, and microbiology of pulmonary superinfections and outcomes

Characteristics	Total influenza pneumonia (38)			Influenza CAP (29)		
	< 724 lymphocytes/μl (*n* = 23)	> 724 lymphocytes/μl (*n* = 15)	*p* value*	< 724 lymphocytes/μl (*n* = 20)	> 724 lymphocytes/μl (*n* = 9)	*p* value
Age, years	71 (57–80)	76 (66–80)	0.681	70 (58–78)	78 (66–80)	0.681
Male sex	6 (26%)	8 (53%)	0.291	4 (20%)	4 (44%)	0.188
N/L ratio	*14.8 (9.4–19.3)*	*4.9 (1.6–8.6)*	*< 0.001*	*15.5 (12.9–30.6)*	*6.8 (3.3–7.8)*	*< 0.001*
Current smoking	17 (74%)	6 (40%)	0.098	*15 (75%)*	*3 (33%)*	*0.034*
Alcohol abuse	1 (4%)	0 (0%)	0.413	1 (5%)	0 (0%)	0.502
Corticosteroid	1 (4%)	1 (7%)	0.754	1 (5%)	1 (11%)	0.754
Influenza virus CAP	20 (87%)	9 (60%)	0.368	–	–	–
Influenza virus HAP	3 (13%)	6 (40%)	0.656	–	–	–
Comorbidity						
Cardiovascolar	18 (78%)	10 (67%)	0.428	16 (80%)	5 (56%)	0.188
Neurologic	7 (30%)	6 (40%)	0.543	6 (30%)	4 (44%)	0.470
Psychiatric	2 (9%)	0 (0%)	0.240	2 (10%)	0 (0%)	0.334
Gastroenteric	4 (17%)	6 (40%)	0.122	2 (10%)	3 (33%)	0.135
COPD	*8 (35%)*	*1 (7%)*	*0.046*	7 (35%)	1 (11%)	0.188
Autoimmune	3 (13%)	2 (13%)	0.979	3 (15%)	1 (11%)	0.776
Nephrologic	6 (26%)	6 (40%)	0.367	3 (15%)	4 (44%)	0.096
Neoplastic	2 (9%)	1 (7%)	0.820	2 (10%)	1 (11%)	0.936
Metabolic	10 (43%)	3 (20%)	0.136	9 (45%)	2 (22%)	0.245
Genetic	1 (4%)	2 (13%)	0.315	1 (5%)	1 (11%)	0.561
Pregnancy	0 (0%)	1 (7%)	0.209	0 (0%)	1 (11%)	0.138
Diabetes	6 (26%)	3 (20%)	0.666	5 (25%)	2 (22%)	0.864
Charlson comorbidity index	5 (2–7)	4 (2–5)	0.209	5 (2–7)	4 (2–5)	0.209
Influenza type						
A	13 (57%)	12 (80%)	0.136	8 (40%)	6 (80%)	0.050
A-H1N1	10 (43%)	3 (20%)	0.135	3 (15%)	3 (33%)	0.276
B	0 (0%)	0 (0%)	1.000	0 (0%)	0 (0%)	1.000
Severity						
ICU	*12 (52%)*	*0 (0%)*	*0.002*	5 (25%)	0 (0%)	0.105
SOFA score	*3 (2–4)*	*1 (1–2)*	*0.004*	*3 (2–4)*	*1 (1–2)*	*0.013*
No oxygen support	1 (4%)	3 (20%)	0.124	*1 (5%)*	*7 (78%)*	*< 0.001*
NIV	4 (17%)	1 (7%)	0.339	4 (20%)	1 (11%)	0.559
IMV	*8 (35%)*	*0 (0%)*	*0.031*	*7 (35%)*	*0 (0%)*	*0.045*
ECMO	2 (9%)	0 (0%)	0.240	2 (10%)	0 (0%)	0.334
Pulmonary superinfection						
Total superinfection	*7 (30%)*	*0 (0%)*	*0.021*	6 (30%)	0 (0%)	0.069
*A. baumannii*, *P. jiroveci*	1 (4%)	0 (0%)	0.413	1 (5%)	0 (0%)	0.502
*A. baumannii*	1 (4%)	0 (0%)	0.413	1 (5%)	0 (0%)	0.502
*MRSA*, *S. maltophila*, *A. fumigatus*	1 (4%)	0 (0%)	0.413	1 (5%)	0 (0%)	0.502
*C. striatum*	1 (4%)	0 (0%)	0.413	1 (5%)	0 (0%)	0.502
*A. fumigatus*	3 (13%)	0 (0%)	0.413	2 (10%)	0 (0%)	0.334
Outcome						
LOS, days	20 (11–40)	22 (11–45)	0.633	20 (11–39)	12 (10–22)	0.633
Mortality	7 (30%)	2 (13%)	0.411	6 (30%)	1 (11%)	0.277

Data are presented as median (interquartile range (IQR) 25–75%) for continuous variables or as simple frequencies (*n*) and percentages for categorical variables

*N/L ratio* neutrophils/lymphocytes ratio, *CAP* community-acquired pneumonia, *HAP* hospital-acquired pneumonia, *COPD* chronic obstructive pulmonary disease, *ICU* intensive care unit, *SOFA score* Sequential Organ Failure Assessment score, *NIV* non-invasive ventilation, *IMV* invasive mechanical ventilation, *ECMO* extracorporeal membrane oxygenation, *LOS* length of stay

*For comparisons between groups, Fisher’s exact test was used for dichotomous variables, the χ^2^ test was used for non-ordered categorical variables and the Mann-Whitney test was used for continuous variables

In our experience, although we evaluated a small sample, a more severe course of a disease might be expected in episodes of L-IP. Under these circumstances, even otherwise immunocompetent patients seem to be at increased risk for opportunistic pulmonary superinfections. Based on the abovementioned, L-IP would require close clinical monitoring for these potentially fatal infectious complications possibly including anti-*Aspergillus* prophylaxis.

## Data Availability

All data are available on request from the corresponding author.
